# Clinical Phenotypes and Age-Related Differences in Presentation, Treatment, and Outcome of Heart Failure with Preserved Ejection Fraction: A Vietnamese Multicenter Research

**DOI:** 10.1155/2021/4587678

**Published:** 2021-01-15

**Authors:** Ngoc-Thanh-Van Nguyen, Diep Tuan Tran, Pham Le An, Sy Van Hoang, Hoai-An Nguyen, Hoa Ngoc Chau

**Affiliations:** ^1^Division of Cardiology, Internal Medicine Department, University of Medicine and Pharmacy at Ho Chi Minh city, Ho Chi Minh city 700 000, Vietnam; ^2^Cardiology Department, Nhan Dan Gia Dinh Hospital, Ho Chi Minh city 700 000, Vietnam; ^3^Outpatient Department, University Medical Center, Ho Chi Minh city 700 000, Vietnam; ^4^University of Medicine and Pharmacy at Ho Chi Minh city, Ho Chi Minh city 700 000, Vietnam; ^5^Family Physician Training Center, University of Medicine and Pharmacy at Ho Chi Minh city, Ho Chi Minh city 700 000, Vietnam; ^6^Cardiology Department, Cho Ray Hospital, Ho Chi Minh city 700 000, Vietnam

## Abstract

**Background:**

Heart failure with preserved ejection fraction (HFpEF) is a rising health problem with heterogeneous presentation and no evidence-based treatment. While Southeast Asia reported the highest mortality and morbidity among Asian population, little is known about the Vietnamese population, including patient characteristics, prescribing pattern and mortality rate.

**Methods:**

We conducted an observational study on 477 patients diagnosed with HFpEF from seven hospitals in Southern Vietnam from January 2019 to December 2019.

**Results:**

Mean age was 67.6 (40.9% < 65 years). 62.3% were female. 82.4% were diagnosed within 5 years. Dyspnea, congestion, and hypoperfusion on admission were noted in 63.9%, 48.8%, and 4.6% of the patients, respectively. Median ejection fraction was 63%. Valvular heart disease (VHD) was the leading cause of heart failure (35.9%). 78.6% had at least two comorbidities, mostly hypertension (68.6%). 30.6% of the patients were hospitalized, with a median stay of 7.0 (4.0–10.0) days and inhospital mortality of 4.8%. Older patients (≥65 years) were more likely to be females (OR = 1.52); had multimorbid conditions (OR = 3.14), including hypertension (OR = 4.28), diabetes (OR = 1.73), coronary artery disease (CAD) (OR = 2.50), dyslipidemia (OR = 1.94), and chronic kidney disease (OR = 2.44); and were more frequently prescribed statin (OR = 3.15). Younger individuals (<65 years) were associated with higher mineralocorticoid antagonist uptake (OR = 0.52) and VHD (OR = 0,40). Prescription rate for renin-angiotensin-aldosterone system inhibitor, beta blocker, mineralocorticoid antagonist, and loop diuretic was 72.5%, 59.1%, 43.0%, and 60.6%, respectively. Four phenotypes were identified, including the lean/elderly/multimorbid; congestive/metabolic; CAD-induced; and younger/atrial fibrillation (AF)/VHD. The novel phenotype “younger/AF/VHD” exhibited high symptom burden and poor functional capacity despite being the youngest and least multimorbid. The “lean/elderly/multimorbid” phenotype demonstrated the highest symptom severity and inhospital mortality.

**Conclusions:**

Our research highlights a younger, predominantly female population with high disease burden. The four novelly identified phenotypes provide contemporary and pragmatic insights into a phenotype-guided approach, exclusively targeting the Vietnamese population.

## 1. Introduction

Heart failure, a rapidly growing public health concern, is taking center stage worldwide [1, 2]. As many patients are reaping benefits from life-saving interventions, more are living with heart failure. The 2016 Heart Failure Guideline of European Society of Cardiology classified heart failure into three categories based on ejection fraction (EF): preserved (≥50%), midrange (40−<50%), and reduced (<40%) [3]. While heart failure with reduced ejection fraction (HFrEF) has seen dramatic transformation with improved mortality and functional capacity, treatment dilemma persists in heart failure with preserved ejection fraction (HFpEF).

Traditionally considered as diastolic heart failure, HFpEF is now proven to have distinct phenotypes, etiologies, and outcomes [4–11]. In developed countries, prevalence of HFpEF is rising, accounting for more than 50% of heart failure [12, 13]. Though patients with HFpEF have a lower risk of death compared with HFrEF, the absolute mortality is high and expanding, heralding a global pandemic, especially in Asia, which accommodates more than 50% of the world population [14, 15]. Exponential population growth, aging baby boomers, and rapid epidemiological transition with clustering of risk factors leave Asians extremely vulnerable to HFpEF, which is associated with old age and high comorbidity [14, 16]. Unfortunately, Asian patients were often either underrepresented in global trials or restricted to regional multicenter study. Conflicting data exists regarding the prevalence, presentation, and mortality, indicating a heterogeneous profile across geographical and ethnic compositions of Asia [16–18]. HFpEF contributed more than 50% of heart failure in Japan and Hong Kong, whereas it only represented one-fifth in ASIAN-HF trial [17, 19]. One-year mortality ranged from 2.9% in South Asia to 10.3% in South East Asia, with an overall of 5.4% in 11 Asian countries [18].

Lack of evidence-based treatment and diverse phenotypes remain challenging issues in HFpEF management. So far, the approach is mostly individualized and heavily focused on phenotypes and comorbidities as presenting features [2, 11, 20, 21]. While the pathophysiology-based phenotyping appeared to be a promising approach, its clinical application is restricted by the mixed-mechanism nature of HFpEF [2]. Another pragmatic perspective is to focus on clinical variables, such as comorbidity, which were not only easily spotted by physicians but also associated with different long-term outcomes [2, 21]. As common phenotypes were observed across population, cardiology experts proposed specific treatment approach and distinct therapeutic response for those frequently presented phenotypes [2, 20, 21]. Yet, slight phenotype variations existed among regional and ethnic groups, calling for more local research on HFpEF patients [1, 18]. Most large-scale, cross-border HFpEF registries exclude Vietnam, home to about 100 million residents [18, 22]. In addition, nationwide social and racial disparity warrants the need of multicenter patient enrolment. We therefore conducted the first HFpEF multicenter study in Vietnam to identify clinical phenotypes, as well as age-related differences in patients' characteristics, treatment pattern, and inhospital mortality rate.

## 2. Materials and Methods

This study was part of the Heart Failure Initiative by University of Medicine and Pharmacy at Ho Chi Minh City. Seven enrolment sites in Southern Vietnam were included: three teaching hospitals (Cho Ray Hospital, Nhan Dan Gia Dinh Hospital, and University Medical Center), two heart centers (Heart Institute in Ho Chi Minh City and Tam Duc Heart Hospital), and two general hospitals (Go Vap District Hospital and Thu Duc District Hospital). These recruitment sites have cardiology expertise and experience in managing a wide range of cardiovascular diseases, including heart failure. Medical ethical approval was obtained prior to data collection from the Committee of Ethics of University of Medicine and Pharmacy at Ho Chi Minh City and appropriate body at each site. The study adhered to principles of medical research laid down in the Declaration of Helsinki. Informed consent was obtained in all participants.

We admitted all consecutive Vietnamese patients diagnosed with HFpEF who attended either inpatient wards or outpatient clinics from January 2019 to December 2019.

### 2.1. Inclusion Criteria

HFpEF was defined as satisfying all three criteria:

(1) EF ≥50%
(2) One of the following criteria:
  - Previously documented NT-proBNP ≥ 450 pg/ml if < 50 year, ≥ 900 pg/ml if 50−<75 year, and ≥1800 pg/ml if ≥ 75 year
  - Diagnosed with heart failure using Framingham criteria [23], and a previously documented NT-proBNP ≥450 pg/ml
(3) Diagnosed as HFpEF by a trained cardiologist at each enrolment site

### 2.2. Exclusion Criteria

Exclusion criteria included the following:

1. End-stage renal or hepatic disease
2. Prior documented EF <50%
3. Takotsubo disease, hypertrophic cardiomyopathy, cardiac amyloidosis, cardiac sarcoidosis, peripartum cardiomyopathy, chemotherapy-induced cardiomyopathy, and constrictive pericarditis
4. Life expectancy <1 year due to noncardiac etiologies
5. Pregnancy or lactation
6. Concurrent enrolment in any other trial

Information on demographic features, medical and behavioral history, clinical symptoms, and functional status was collected through direct interview and physical examination. Risk factors and comorbidities were either taken from electrical medical records or newly identified, which included but were not limited to coronary artery disease (CAD), hypertension (HTN), atrial fibrillation/atrial flutter (AF), diabetes mellitus (DM), chronic kidney failure (CKD), smoking, obesity, peripheral artery disease (PAD), cerebral vascular accident (CVA), obstructive pulmonary disease, and cancer.

Heart failure etiology was determined by the attending cardiologists at each enrolment site. CAD was defined as having a positive angiogram or noninvasive tests (MSCT coronary angiography, SPECT/PET, dobutamine stress echocardiogram, and exercise stress test). HTN was defined as persistent elevation of blood pressure beyond 140/90 mmHg or currently on antihypertensive medications. CVA and PAD were diagnosed with a positive angiogram, >50% stenosis on arterial Doppler for lower extremity (for PAD), positive brain CT scanner/MRI (for CVA), or history of intervention. DM was diagnosed using the 2019 ADA guideline or recorded use of antiglycemic drugs [24]. Cutoff point for obesity was in keeping with Asia-Pacific population at 25 kg/m^2^ [25]. CKD was defined as a sustained drop of estimated glomerular filtration rate to <60 ml/min using the 2012 CKD-EPI equation or documented structural abnormalities persisting for more than three months [26]. Obstructive pulmonary disease included asthma and chronic obstructive pulmonary disease (COPD) and were diagnosed with positive pulmonary function test, prior diagnosis, or current treatment. Congestion and hypoperfusion are defined according to the 2016 European Society of Cardiology guideline on heart failure. Congestion referred to pulmonary congestion, orthopnea/paroxysmal nocturnal dyspnea, peripheral (bilateral) oedema, jugular venous dilatation, congested hepatomegaly, gut congestion, ascites, and hepatojugular reflux [3]. Hypoperfusion referred to cold sweaty extremities, oliguria, mental confusion, dizziness, and narrow pulse pressure [3]. Echocardiogram and electrocardiogram were interpreted by experienced cardiologists. Data on medical prescription, patient education, and vitals were collected at first contact. All patients were required to have a documented NT-proBNP meeting the inclusion criteria. During the conduction of this study, stable patients at the outpatient department were not obliged to reperform another test. Body mass index was calculated by body weight (kg) divided by height square (m^2^), with patients wearing light clothes and standing on barefoot during measurement.

Data were reported as either mean ± standard deviation (SD) for normally distributed variables or median (interquartile range, IQR) for skewed variables. Categorical variables were displayed as percentage. *T*-test or Wilcoxon rank sum test was used for continuous variables. Chi square or Fisher exact test was used for dichotomous or categorical variables. Odds ratios were presented with 95% interval. All two-tailed tests with a *p* value of <0.05 were considered statistically significant. Analysis was conducted using IBM SSSS Statistics 26.

Using the poLCA package in *R*, we performed the latent class analysis (LCA) to identify the clinical phenotypes of HFpEF. The number of phenotype parameter was incrementally updated after each iteration until it reached the minimum Bayesian information criterion (BIC). Participants were categorized into groups with similarities based on age, sex, body mass index, dyslipidemia, HTN, DM, CAD, VHD, AF, CKD, smoking, dyspnea, congestion, hypoperfusion, and NYHA class.

## 3. Results

### 3.1. Clinical Phenotypes

**Figure 1:**
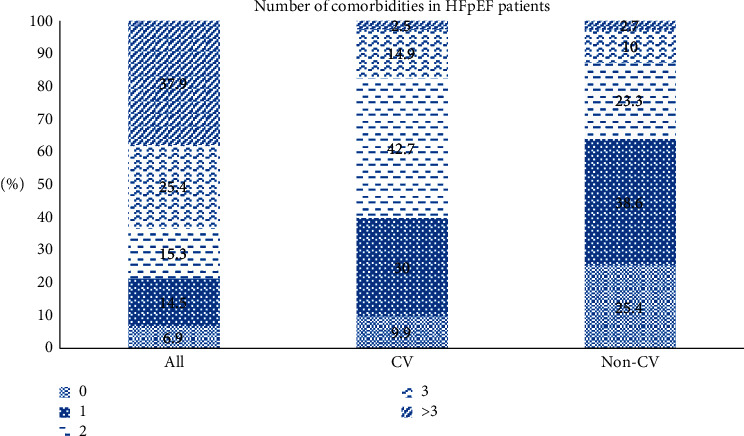
Pie chart showing the number of comorbidities: total, cardiovascular (CV), and noncardiovascular.

During the study period, a total of 509 patients met the predefined HFpEF criteria. Among them, we were able to obtain 100% information on the 15 variables used in LCA from 477 patients. Thirty-two patients were excluded due to inadequate information on BMI (*n* = 5); smoking (*n* = 8); HF etiology (*n* = 2); AF type (*n* = 3); CKD (*n* = 4); congestion (*n* = 6); hypoperfusion at admission (*n* = 4). Mean age of patients was 67.6 ± 14.4 years (40.9% < 65 years). Females comprised of 62.3% of the population. 82.4% of individuals were diagnosed within five years. A history of hospitalization in the preceding 12 months and current hospitalization were reported in 41.1% and 30.6% of patients. Congestion, dyspnea, and hypoperfusion were present in 48.8%, 63.9%, and 4.6%, respectively. The most common cause of heart failure was valvular heart disease (35.9%). 78.6% of patients had at least two concurrent diseases, with cardiovascular more than noncardiovascular comorbid conditions (Figure 1). 59.9% of patients had at least two cardiovascular comorbidities, while 74.6% of patients reported noncardiovascular comorbid disease. As NT-proBNP was not an obligation in stable patients, 59.3% of patients had on-the-spot NT-proBNP testing during the conduction of this study. Median NT-proBNP was 1951 (2955) pg/ml. The most frequently prescribed medication was statin (72.5%) and renin–angiotensin–aldosterone inhibitors (RAASi) (72.5%). RAASi intolerance was noted in 5.5% of patients, most often due to cough in case of angiotensin-converting enzyme inhibitors (ACEi) and hypotension in case of angiotensin II receptor blockers (ARB). The most common contraindication for MRA was worsening renal function. In hospitalized patients, inhospital mortality rate was 4.7% with a median duration of 7.0 (4.0–10.0) days.

Using the predefined LCA function in poLCA package of *R*, we identified four different phenotypes as described in Table 1. Phenotype 1 (*n* = 64) was mostly elderly lean females with worst symptom severity and functional capacity and highest comorbid burden especially HTN, DM, AF, and CKD. These patients had the highest rate of hospitalization and inpatient mortality. Phenotype 2 (*n* = 201) consisted of mostly obese, nonsmoking females with significant dyslipidemia, HTN, CAD, and DM. Congestion and high uptake of RAS inhibitor and BB were notable. Phenotype 3 (*n* = 88) included least congestive, smoking male patients, whose CAD was the main cause for HFpEF. This population reported high rate of BB intake. Phenotype 4 (*n* = 124) was comprised of a young population with least disease burden except for AF and VHD. High rate of dyspnea and high uptake of loop diuretics and MRA were noted.

NT-proBNP was available for the majority of patients with phenotype 1 (70.3%), who had highest hospital admission (82.8%), compared with the other three phenotypes (around 50%). Their levels were approximately twice those of phenotypes 2, 3 and 4, reflecting a worse prognosis.

As HFpEF was characterized by multicomorbidity, we compared the inhospital mortality rate in four phenogroups according to the number of total diseases. No significant differences were observed in terms of disease burden (*p*=0.2). Highest mortality was noted in those with two to three diseases, whereas those with zero to one disease survived.

**Figure 2:**
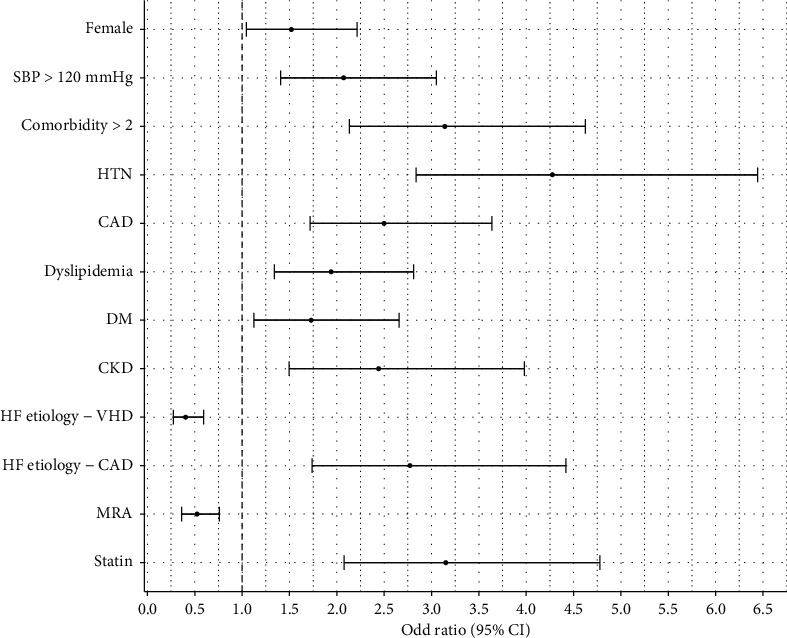
Forest plot depicting association between patient characteristics, comorbidity, treatment, and odds of being age ≥65 versus <65. CAD: coronary artery disease; HTN: hypertension; AF: atrial fibrillation/flutter; DM: diabetes mellitus; CKD: chronic kidney disease; VHD: valvular heart disease; SBP: systolic blood pressure; MRA: mineralocorticoid antagonist.

We also compared the elder (≥65 years) and younger (<65 years) patients (Figure 2). Elder individuals were more likely to be females (OR = 1.52, 95% CI 1.04–2.21), having more comorbidities (OR = 3.14, 95% CI 2.13–4.63), higher systolic blood pressure (OR = 2.07, 95% CI 1.41–3.05), and more frequently prescribed statin (OR = 3.15, 95% CI 2.08–4.78). They were more prone to HTN (OR = 4.28, 95% CI 2.84–6.44), DM (OR = 1.73, 95% CI 1.12–2.66), dyslipidemia (OR = 1.94, 95% CI 1.34–2.81), CKD (OR = 2.44, 95% CI 1.50–3.98), and CAD (OR = 2.50; 95% CI 1.72–3.64). Younger patients were associated with VHD-induced HFpEF (OR = 0.40, 95% CI 0.27–0.59) and had higher MRA prescription (OR = 0.52, 95% CI 0.36–0.76) (Figure 2). Despite the age difference, there were no dissimilarities between the two groups in terms of symptom burden (congestion, dyspnea, and hypoperfusion), functional capacity (NYHA III-IV), hospitalization, and loop diuretic treatment (*p* < 0.05).

## 4. Discussion

Our study was the first multicenter HFpEF trial in Vietnam, with participation from a wide range of clinical practice (primary and tertiary care, specialized and general hospitals, and inpatient and outpatient settings). We described a predominantly female population, with mean age of 67.5 and median ejection fraction of 63%. Since HFpEF was more common in the elderly, and 40.9% of our population were <65 years old, we compared the age-related differences in patients' characteristics and inhospital mortality between younger and elder (≥65 years) patients. We then contrasted our result with two studies, the first one pooled data from the TOPCAT, I-PRESERVED and CHARM-Preserved trial, and the second one analyzed the HFpEF subgroup of ASIAN-HF Registry [27, 28]. In both studies, obesity and diabetes were more common in the younger population, while atrial fibrillation, heart failure hospitalization and mortality were associated with the elder group [27, 28]. Yet, in our study, both obesity and diabetes were more common in the elder group, and no differences were observed with regard to functional capacity, symptom burden, prior hospitalizations in the preceding 12 months, and inhospital mortality. Specifically, the younger group was distinctly associated with VHD. These variations should be interpreted in the light of regional diversity in the clustering of morbidities in HFpEF (Table 2). Compared with patients from Asia (ASIAN-HF), Japan (JASPER), Europe (I-PRESERVED), and other parts of the world (TOPCAT), our patients demonstrated lowest BMI, DM, and CKD [17, 29–31]. Furthermore, VHD was the most common cause of HFpEF in our study (37.1%), whilst CAD was the leading etiology in TOPCAT trial (59%) [31]. Variety in clustering of comorbidities and etiologies can be associated with diversity in age-related differences in presentations and outcomes.

As HFpEF was a heterogeneously multimorbid syndrome, current approach shifts the focus on the clustering of clinical presentation (phenotype) rather than individual condition. While HFpEF phenotypes can vary among studies, some phenotypes were consistent in most trials [5, 20, 32]. They included but were not restricted to (1) elderly/multimorbidity phenotype; (2) CAD-induced phenotype; (3) right heart failure/pulmonary HTN phenotype; (4) metabolic/“garden-variety” phenotype [5, 8, 17, 20, 32–34]. In our analysis, four distinct phenotypes were identified, three of which resembled those previously described [8, 17, 33, 34]. Notably, one novel phenotype emerged, exhibiting a unique clustering of clinical features.

In our study, phenotype 1 was consistent with the “elderly/multimorbid” phenotype. They were the oldest, leanest, and having the most symptoms and disease burden. On average, each patient had four concurrent diseases, mostly HTN, CAD, AF, CKD, and DM. Congestion, dyspnea, and hypoperfusion was noted in 98.4%, 95.3%, and 29.7%, respectively. Highest NT-proBNP levels were noted in this group at 4005 (6165) pg/ml. These figures partially elaborated a high percentage of inhospital care (82.8%) and mortality (9.4%). Pooled data from the I-PRESERVED, CHAMR-Preserved, and ASIAN-HF reported similarly poor outcome in “elderly/multimorbid” phenotype with preponderant AF, CKD, CAD, HTN, and DM [17, 34]. The higher the comorbidity burden, the higher the mortality rate [35].

Phenotype 2 demonstrated the classic metabolic HFpEF with pronounced obesity, dyslipidemia, HTN, DM, and congestion. This metabolic phenotype was traditionally associated with obesity, especially in the American population. However, recent European and Asian studies illustrated a nonobese metabolic phenotype [17, 33, 34]. This is of great importance in Asian HFpEF, who had leaner body composition yet higher diabetes prevalence than Western population (Table 2) [36]. In our study, HTN and DM were mostly predominant in phenotypes 1 (17.2% obese) and 2 (40.3% obese). These two phenotypes were the oldest and most multimorbid and were often prescribed loop diuretics (Table 1). The obese phenotype 2 revealed the highest rate of ARB, BB, and statin intake, whereas the lean phenotype 1 was the most symptomatic, requiring hospitalization and loop diuretic prescription (Table 1). Smoking rate was 30.3% in phenotype 1 as opposed to 0% in phenotype 2.

Phenotype 3 was characteristic of the CAD-induced phenotype. 97.7% of patients were males, with a soaring smoking rate of 83.0%. CAD was the most common cause for HFpEF (43.2%), and 21.6% of the patients had undergone revascularization. This phenotype was least likely to be hospitalized (14.8%) and reported the lowest NT-proBNP level (1416 pg/ml). These patients showed the fewest congestion, best functional capacity, and highest sacubitril/valsartan uptake (Table 1).

Most importantly, to the best of our knowledge, our study was the first to describe the novel phenotype of AF and VHD females. This finding stems from the variation in disease prevalence. In developed countries, degeneration was the leading cause of VHD. Yet, in our nation, acute rheumatic fever was still common. Without proper treatment and followup, postrheumatic VHD developed and became clinically overt as early as the fourth or fifth decade [37]. Subsequent atrial derangement occurs, leading to the development of atrial arrhythmia, AF, and eventually heart failure at an earlier time compared with Western patients. As a result, the youngest group (phenotype 4), despite having the least number of comorbidities, reported the highest rate of VHD and AF (79.8% and 62.9%). In contrast, in high-income nations, the youngest phenotype was often associated with obesity, diabetes, and multiple risk factors, whereas the eldest phenotype often showed highest prevalence of AF [7, 19, 27, 33].

Our study provides valuable contributions to the body of literature for two important reasons. First, we conducted the first multicenter study on HFpEF in Vietnam, describing four distinct phenotypes. Among these four phenotypes, three were consistent with existing, classic phenotypes (phenotype 1: lean/elderly/multimorbid, phenotype 2: congestive/metabolic, and phenotype 3: CAD-induced), and one was newly identified for the first time (phenotype 4: youngest/VHA/AF). Different clustering of comorbidities, functional capacity, and inhospital mortality observed in each phenotype shed some light on phenotype-specific approach. In individual comorbidity level, some of our comorbid patterns were not dissimilar to the Asian population (leaner body composition; younger age of onset), and some were more comparable with Western counterparts (CAD and AF prevalence) (Table 2). Taken together, these facts suggest a mosaic and unique disease spectrum, reflecting the transition from infectious to noncommunicable diseases.

Second, our study proposes an essential implication in everyday practice. From a clinical perspective, the critical focal point of care was high-risk patients, especially the elderly with multimorbidity (phenotype 1). This is of greater importance in HFpEF population, as the number of disease was positively associated with one-year mortality [38]. In our study, although the maximum mortality was noted in hospitalized patients from phenotype 1 (lean/elderly/multimorbid) (Table 1), the highest mortality was observed in those with two to three diseases and not those with ≥4 diseases (Table 3). This finding suggested that multimorbidity alone could not justify the high mortality rate of phenotype 1. Therefore, physicians should take appropriate precaution against this vulnerable population, employing a comprehensive approach for better treatment outcome.

## 5. Limitations

Our study was subjected to site and individual selection bias. As diastolic stress tests and right heart catheterizations were not available at all sites, site investigators reserved the right to include or exclude cases of uncertainty. Furthermore, the development of HFpEF may be multifactorial in origin, whereas, according to our protocol, only one was selected as the main etiology by site investigators. This approach simplified and eased the analysis of HF etiologies at the cost of missing reports on possible coetiologies. Our study was also prone to selection bias, as we excluded patients with end-stage liver/renal disease, amyloidosis, or sarcoidosis, limiting the representativeness of the research in terms of clinical practice. Due to the overburdened patient volume in participating hospitals, not all HFpEF patients agreed to participate in the study, as this would lengthen the duration of examination and consultation. To partially counterbalance the selection bias, our prime investigators at each site, who were acting as chief of heart failure clinics, continuously monitored the patient volume and distribution to ensure the representativeness of the study population. Information on smoking, drinking, patient education, employment status, education level, and duration of heart failure was retrieved from the interview alone, leading to potential recall bias. Another concern was VHD, as some studies separated these patients from the overall HFpEF population due to their specific clinical findings and treatment options. While the majority of HFpEF etiologies were VHD and hypertensive [38], VHD-induced HFpEF remained a controversial issue. However, as VHD was identified as the major etiology in our studies, and postrheumatic VHD persisted as a challenging problem in Vietnam, we decided to include them in the final analysis. By doing so, we aimed to provide the literature with a comprehensive picture of Vietnamese HFpEF, as well as raising the awareness of an easily overlooked phenotype 4, who, despite being the youngest and least multimorbid, reported the second highest rate of symptom and hospitalization. Finally, while we demonstrated variation in inhospital mortality among phenotypes, data on long-term outcome were lacking. At the present time, we are following up patients to evaluate the association between specific cardiovascular outcomes and clinical phenotypes. More well-designed, multicenter research should be conducted to further investigate the application of phenotyping patients in guiding treatment.

## 6. Conclusions

These first multicenter data from Vietnam highlight a younger population with a significant disease burden, various clinical presentation, and poor functional status. Our study underpinned a unique and diverse phenotype spectrum, from the younger VHD/AF female to the leaner/multimorbidity elderly and from the congestive/metabolic females to the least congestive/CAD-induced males. These phenotypes with specific clinical patterns and inhospital mortality rate necessitate the need for future research on phenotype-guided approach, specifically targeting Vietnamese population.

## Figures and Tables

**Table 1:** Four clinical phenotypes of patients diagnosed with HFpEF.

	All	Phenotype 1	Phenotype 2	Phenotype 3	Phenotype 4	Adjusted *P* value
N	477	64	201	88	124	—
*Patients' characteristics*						
Age (year)	67.6 (14.4)	74.0 (12.4)	71.9(10.7)	67.7 (13.6)	57.4 (15.9)	<0.001^*∗*^
Male (%)	37.7	42.2	15.4	97.7	29.0	<0.001^*∗*^
Obesity (%)	28.7	17.2	40.3	31.8	13.7	<0.001^*∗*^
Smoking (%)	22.0	31.3	0	83.0	9.7	<0.001^*∗*^
EF (%)	63 (13/0)	61 (12.5)	65 (13.0)	60 (9.3)	64 (10.3)	0.008^*∗*^
SBP (mmHg)	120 (20.0)	128 (34.5)	120 (20.0)	120 (30.0)	110 (20.0)	<0.001^*∗*^
DBP (mmHg)	70 (20.0)	73 (22.0)	70 (20.0)	70 (20.0)	66.5 (11.7)	0.005^*∗*^
Resting HR (bpm)	80 (18.0)	81 (25.0)	78 (17.5)	79 (18.0)	81 (21.0)	0.132
NYHA III-IV (%)	28.3	100.0	8.5	6.8	38.7	<0.001^*∗*^
NT-proBNP available	53.1%	70.3%	43.3%	55.7%	58.1%	—
NT-proBNP (pg/ml)	1951 (2955)	4005 (6165)	1891 (2892)	1416 (2724)	1924 (2245)	<0.001^*∗*^

*Comorbidities*						
Dyslipidemia (%)	54.3	51.6	79.1	60.2	11.3	<0.001^*∗*^
Hypertension (%)	68.6	95.3	95.0	75.0	7.3	<0.001^*∗*^
Diabetes (%)	26.6	35.9	39.3	20.5	5.7	<0.001^*∗*^
CAD (%)	50.7	81.3	62.7	56.8	11.3	<0.001^*∗*^
Prior revascularization (%)	10.3	12.5	11.0	21.6	0	<0.001^*∗*^
AF (%)	38.8	42.2	26.4	30.7	62.9	<0.001^*∗*^
CKD (%)	21.6	43.8	23.4	15.9	11.3	<0.001^*∗*^
CVA (%)	10.7	14.1	10.0	14.8	7.3	0.286
Asthma/COPD (%)	4.6	9.4	3.0	10.2	0.8	0.002^*∗*^
Cancer (%)	2.3	1.6	2.5	3.4	1.6	0.807
Number of comorbidities	3 (2)	4 (2)	4 (1)	3 (2)	1 (2)	<0.001^*∗*^

*HFpEF etiologies*						
CAD-induced (%)	25.4	34.4	28.9	43.2	2.4	<0.001^*∗*^
HTN-induced (%)	22.6	34.4	33.3	20.5	0.8	<0.001^*∗*^
DCM-induced (%)	2.5	1.6	2.0	5.7	1.6	0.293
VHD-induced (%)	35.9	26.6	20.9	14.8	79.8	<0.001^*∗*^
Other causes (%)	9.9	3.1	8.5	13.6	12.9	0.099

*Symptoms*						
Dyspnea (%)	63.9	95.3	59.2	62.5	56.5	<0.001^*∗*^
Congestion (%)	48.8	98.4	45.3	34.1	39.5	<0.001^*∗*^
Hypoperfusion (%)	4.6	29.7	0	2.3	0.8	<0.001^*∗*^
Inpatient setting						—
Inpatient care (%)	30.6	82.8	15.4	14.8	39.5	<0.001^*∗*^
Inhospital mortality (%)	4.8	9.4	3.2	0	2.0	0.342

*Treatment pattern*						
ACEi	27.0	31.3	31.3	23.9	20.2	0.132
ARB	45.5	32.8	55.7	48.9	33.1	<0.001^*∗*^
Sacubitril/valsartan	1.3	1.6	1.0	3.4	0	0.132
BB	59.1	40.6	69.2	62.5	50.0	<0.001^*∗*^
MRA	43.0	42.2	33.8	40.0	60.5	<0.001^*∗*^
Loop diuretic	60.6	79.7	50.8	53.4	71.8	<0.001^*∗*^
Statin	72.5	81.3	87.6	84.1	35.5	<0.001^*∗*^

^*∗*^Significant after the Benjamini–Hochberg procedure (false discovery rate 0.05). SBP: systolic blood pressure; DBP: diastolic blood pressure; HR; heart rate; NYHA: New York Heart Association, NT-proBNP: N-terminal pro-brain natriuretic peptide; PND: paroxysmal nocturnal dyspnea; CAD: coronary artery disease; HTN: hypertension; AF: atrial fibrillation/flutter; DM: diabetes mellitus; CKD: chronic kidney disease; DCM: dilated cardiomyopathy; COPD: chronic obstructive pulmonary disease; MI: myocardial infarction; ACEi: angiotensin-converting enzyme inhibitor; ARB: angiotensin II receptor blocker; BB: beta blocker; MRA: mineralocorticoid antagonist.

**Table 2:** Body mass index and comorbidity rates of our study compared with ASIAN-HF Registry, JASPER, TOPCAT, and I-PRESERVE trials.

Variables	Our	ASIAN-HF	JASPER	TOPCAT	I-PRESERVE
N	477	1204	535	3445	4133
BMI (Kg/m^2^)	22.8	27.1	23.9	32	30

*History*					
CAD (%)	50.7	29.5	27.7	59	48
HTN (%)	68.6	71.2	77.2	91	88
AF (%)	38.8	28.6	61.5	35	29

*Non-CV comorbidities*					
Dyslipidemia (%)	54.3	—	42.2	60	44
DM (%)	26.6	45	38.1	32	28
CKD (%)	21.6	50.2	50.8	39	31

CAD: coronary artery disease; HTN: hypertension; AF: atrial fibrillation/flutter; DM: diabetes mellitus; CKD: chronic kidney disease; BMI: body mass index.

**Table 3:** Inhospital mortality according to number of comorbid diseases in four phenotypes.

Number of comorbidity	All inhospital patients (*n* = 146) (%)	Phenotype 1 inhospital patients (*n* = 53) (%)	Phenotype 2 inhospital patients (*n* = 31) (%)	Phenotype 3 in-hospital patients (*n* = 13) (%)	Phenotype 4 in-hospital patients (*n* = 49) (%)
0–1	0	0	0	0	0
2–3	7.9	15.4	0	0	20
≥4	3.9	3.7	5.5	0	0

## Data Availability

Access to data is restricted as this is a part of the ongoing Heart Failure Initiative from University of Medicine and Pharmacy at Ho Chi Minh City. Follow-up is being carried out by investigators in charge at specific sites.
